# From digital twins to clinically trustworthy twins: a clinical-claim-based validation framework for personalized digital health

**DOI:** 10.3389/fdgth.2026.1908794

**Published:** 2026-08-03

**Authors:** Alexandre Vallée

**Affiliations:** Department of Epidemiology and Public Health, Foch Hospital, Suresnes, France

**Keywords:** artificial intelligence, calibration, causal inference, digital health, digital twin, epidemiology, implementation science, personalized medicine

## Abstract

Digital twins are increasingly presented as a computational foundation for personalized and preventive medicine, because they promise to integrate multimodal data into dynamic representations of patients, organs, diseases, or care pathways. Yet the translational maturity of medical digital twins remains limited. Many systems labelled as digital twins are still digital models, digital shadows, descriptive simulations, or prediction tools whose clinical claims exceed the evidence provided for calibration, uncertainty, transportability, causal validity, or real-world utility. This Perspective argues that the next bottleneck for digital twins in health is not model complexity but clinical trustworthiness. The main contribution of this Perspective is a claim-to-evidence typology that links the evidentiary burden of a digital twin to the clinical claim it makes, rather than to its computational architecture alone. This approach connects individual-level multimodal modelling with epidemiology, prediction science, causal inference, and implementation science. This article outlines a staged framework that distinguishes descriptive, predictive, counterfactual, interventional, and population-health clinical claims made by systems labelled or proposed as digital twins, each associated with a distinct evidentiary threshold. This article further proposes that validation should integrate verification, calibration, external and temporal validation, uncertainty quantification, fairness assessment, target trial emulation where causal claims are made, and post-deployment monitoring. Without such methodological discipline, digital twins may remain sophisticated but clinically fragile simulations. Conversely, population-calibrated and prospectively evaluated digital twins could become a robust infrastructure for personalized prevention, adaptive treatment, and learning health systems.

## Introduction

Digital twins have become one of the most visible concepts in contemporary digital health. In engineering, a digital twin refers to a dynamically updated virtual representation of a physical system, connected to that system through data flows and used to anticipate behavior, test scenarios, and inform decisions. Translated to medicine, this idea has stimulated expectations that patient-specific models could simulate disease trajectories, forecast risks, optimize treatment strategies, and support preventive care at a level of granularity that conventional clinical prediction models cannot achieve (1–8). The concept has been particularly influential in precision cardiology, oncology, immune-system modelling, digital phenotyping, and hospital operations (1, 6, 7, 9).

However, enthusiasm for digital twins has outpaced the evidentiary standards required for their clinical use. The field currently wavers between two dominant paradigms: historical biophysical modelling (e.g., the Virtual Physiological Human (10)) which prioritizes mechanistic fidelity, and recent deep learning approaches which prioritize data-driven predictive power. Neither paradigm inherently guarantees clinical safety. The term is now applied to heterogeneous artefacts, including static anatomical models, sensor dashboards, virtual patient cohorts, risk scores, mechanistic organ models, and AI-driven decision-support systems. Recent scoping reviews suggest that only a minority of models described as health or human digital twins satisfy core requirements such as personalization, dynamic updating, predictive capability, and decision relevance (11, 12). This definitional expansion is not merely semantic; it affects how evidence is interpreted, how safety is assessed, and how clinicians understand the boundary between visualization, prediction, and intervention. This distinction can be clarified using representation-level taxonomies developed in the broader digital twin literature. A digital model is a digital representation of a physical system without automatic data exchange. A digital shadow is a digital representation automatically updated by one-way data flow from the physical system to the virtual representation, typically supporting monitoring or retrospective analysis. A digital twin implies a higher level of integration, with dynamic updating and bidirectional coupling between the physical and virtual entities, such that information from the virtual representation may inform, adapt, or influence the physical system or its management. These distinctions are well established in engineering and manufacturing taxonomies, but they remain inconsistently applied in healthcare, where dashboards, risk scores, simulations, and decision-support tools are often grouped under the same digital twin label (13, 14). The present framework is therefore complementary to, rather than a substitute for, these representation-level taxonomies. Digital model, digital shadow, and digital twin taxonomies classify systems according to the degree of data exchange, synchronization, and coupling between the physical and virtual entities. The claim-to-evidence framework proposed here classifies the evidentiary burden according to what the system is used to claim clinically. A one-way monitoring system may be best described as a digital shadow and require evidence for data fidelity, reliability, and usability. A system that forecasts future risk makes a predictive claim and therefore requires calibration and external validation. A system that simulates alternative treatment strategies makes a counterfactual claim and requires causal specification. A system that recommends or modifies clinical action makes an interventional claim and requires prospective evidence of safety, utility, and workflow impact (11, 12).

This Perspective does not seek to broaden the digital twin label or to imply that every monitoring dashboard, prediction model, pathway simulator, or decision-support tool should be considered a digital twin. Some systems currently described as digital twins may be more accurately characterized as digital shadows, dynamic prediction models, simulation models, dashboards, or clinical decision-support systems. The pragmatic position adopted here is that, once such systems are labelled or deployed as digital twins, the key question for clinical safety is not the label itself but the claim being made. The evidentiary discipline proposed in this framework therefore comes from matching the validation standard to the clinical claim, rather than from expanding the definition of what counts as a digital twin.

Our framework aims to bridge the gap between computational modelling, causal inference, and implementation science by setting specific evidentiary thresholds for distinct clinical claims. Our previous viewpoints (3, 4) have argued that medical digital twins require epidemiological data, mathematical modelling, and population-level grounding to become clinically meaningful. The present Perspective does not seek to restate that conceptual argument. Its incremental contribution is to translate this principle into a claim-based evidentiary framework. Specifically, this article proposes that the level and type of validation required for a medical digital twin should depend on whether it is used for representation, prediction, counterfactual simulation, clinical intervention, or population-health decision-making. In this framing, population calibration is not the headline claim, but one methodological requirement within a broader architecture of clinical trustworthiness.

### The translational gap: from representational fidelity to clinical claim

Systems labelled or proposed as medical digital twins differ in the clinical claims they make. A descriptive or monitoring claim concerns the representation of a patient, organ, disease state, or care pathway. Its main requirement is fidelity of representation and usability. A predictive claim concerns forecasting an outcome, trajectory, complication, resource use, or treatment response. It therefore requires the same level of transparent reporting, internal validation, external validation, and calibration expected from clinical prediction models (15–17). A counterfactual twin estimates what would happen under alternative interventions or care strategies. It cannot be validated by discrimination or calibration alone, because it makes a causal claim and requires explicit assumptions about confounding, treatment assignment, time zero, eligibility, follow-up, adherence, and competing events (18). An interventional twin goes further by recommending or modifying clinical decisions. It should therefore demonstrate clinical utility, safety, workflow compatibility, and prospective impact (19–22).

This typology matters because the same computational object can be safe as a descriptive model but unsafe as an interventional system. A three-dimensional cardiac model may be appropriate for explanation or procedural planning, but its evidentiary burden increases if it is used to select therapy, alter medication, or prioritize patients. Similarly, a model that accurately reconstructs prior trajectories may not be valid for forecasting future trajectories under treatment. The clinical claim, rather than the technology, should determine the standard of evidence. Table 1 proposes a structured mapping between the type of digital twin claim and the minimum validation evidence that should accompany it.

**Table 1:** Evidence requirements according to the clinical claim made by systems labelled or proposed as medical digital twins.

Clinical claim	Primary function	Minimum validation evidence	Methodological anchors	References
Descriptive or monitoring claim	Represents a patient, organ, disease state, or care pathway and may update over time.	Data fidelity, measurement reliability, usability testing, interpretability of displayed states, and assessment of missingness or sensor noise.	Digital twin definitions and scoping reviews; VVUQ principles.	(11, 12, 33)
Predictive claim	Forecasts risk, disease progression, complications, resource use, or treatment response.	Transparent reporting, internal and external validation, calibration-in-the-large, calibration slope, flexible calibration curves, temporal validation, subgroup performance, and decision-curve analysis.	TRIPOD + AI, PROBAST + AI, calibration literature.	(15–17, 23, 47)
Counterfactual claim	Simulates alternative interventions, care strategies, or exposure scenarios.	Explicit causal estimand, target trial specification, confounding control, sensitivity analyses, competing-risk handling, and separation of prediction from causal effect estimation.	Target trial emulation and causal inference standards.	(18, 23)
Interventional claim	Recommends, prioritizes, or modifies clinical decisions in real time or near real time.	Prospective live evaluation, workflow integration, human factors assessment, safety monitoring, net benefit, randomized or quasi-experimental impact evaluation, accountability framework, regulatory classification, and post-market monitoring plan.	DECIDE-AI, SPIRIT-AI, CONSORT-AI, FUTURE-AI, EU AI Act, MDR/IVDR, FDA PCCP.	(19–22, 43–46)
Population-health claim	Informs prevention strategy, resource allocation, screening, surveillance, or health-system planning.	Population calibration, transportability across settings, prespecified fairness metrics, equity assessment, medico-economic evaluation, scalability, and post-deployment monitoring for drift and unintended consequences.	Fairness and drift monitoring literature.	(24, 25, 27, 28, 39–42)

Population calibration does not replace the methodological anchors listed in the table. It defines the empirical reference layer against which the outputs of a digital twin should be judged, particularly when the twin produces longitudinal trajectories, treatment scenarios, or dynamically updated predictions.

The categories in this table should not be read as definitional criteria for what is or is not a digital twin. They describe clinical claims that may be made by systems labelled as digital twins. A conventional prediction model, monitoring dashboard, or simulation tool should not be reclassified as a digital twin solely because it performs one of these functions; however, if such a system is deployed under the digital twin label, its evidentiary requirements should still match the clinical claim it makes.

The most frequent translational error is to treat discrimination as clinical validity. A model may rank patients correctly while providing poorly calibrated absolute risks. In prevention and treatment selection, this is problematic because clinical action depends on risk thresholds, expected benefit, and the balance between harms and benefits. Calibration is therefore not a secondary technical metric; it is a prerequisite for individualized decision-making (17). For digital twins, the challenge is amplified because predictions may be dynamically updated, multimodal, and scenario dependent. A clinically useful twin should therefore provide not only a predicted trajectory, but also uncertainty bounds and a statement of the population to which that trajectory is calibrated.

To illustrate this typology, consider an artificial intelligence model in cardiology. A predictive twin might forecast the absolute risk of heart failure progression based on current biomarkers. It therefore requires the same level of transparent reporting, internal validation, external validation, and calibration expected from clinical prediction models. However, a clinician requires a counterfactual twin to answer: “What would this patient's trajectory be if we initiated a specific therapy today, compared to a placebo?”. This shift makes a causal claim and requires explicit assumptions about confounding, treatment assignment, time zero, eligibility, follow-up, adherence, and competing events. Standard predictive deep learning cannot guarantee this without causal inference methods, such as target trial emulation. Furthermore, an interventional twin goes further by recommending or modifying clinical decisions, for example, by actively routing a patient within a regional care network platform based on real-time disease progression and local facility capacities. It should therefore demonstrate clinical utility, safety, workflow compatibility, and prospective impact.

### Population calibration as an evidentiary layer

This article defines a population-calibrated digital twin as a dynamic, patient-specific model whose predictions and simulated trajectories are constrained by empirical distributions of risk, disease progression, treatment response, and competing events observed in relevant populations. This definition does not oppose individualized modelling to epidemiology. On the contrary, it considers epidemiology as the empirical reference system that prevents individualized simulations from drifting into implausible or non-transportable scenarios (Box 1).

Box 1 **Conceptual notation and practical diagnostics for population calibration.**

The notation below is intended as conceptual scaffolding rather than as a new estimator or identifiability result. Its purpose is to clarify the type of empirical compatibility expected from a population-calibrated digital twin.

More formally, let Hᵢ(t) denote the longitudinal health history of patient i up to time t, including clinical events, treatments, biomarkers, imaging, omics, sensor-derived measures, patient-reported outcomes, and relevant contextual exposures. A patient-specific digital twin can be represented as a dynamic mapping that uses Hᵢ(t), model parameters *θ*, and a candidate future care strategy ā(t:t+*τ*) to generate a probabilistic forecast of future states or outcomes over a horizon *τ*:

Ti(t)=F^i,t^τ(Y|Hi(t),a¯(t:t+τ),θ)

In this formulation, the output of the twin is not a single deterministic trajectory but a distribution of plausible trajectories, with explicit uncertainty around future states, outcomes, and decisions.

A population-calibrated digital twin adds an external empirical constraint. Let P_s denote the intended-use population or clinical setting and let G denote clinically relevant subgroups defined by age, sex, comorbidity, socioeconomic position, ancestry, device type, site, or care pathway. The twin is population-calibrated when its predicted probabilities and simulated trajectories are compatible with observed outcomes in P_s, both overall and within relevant subgroups. For binary or time-to-event outcomes, this requires that predicted risk corresponds to observed risk across the range of predicted probabilities and across subgroups:

$$\mathrm{Pr}[Y(t+\tau )=1\;|\;\hat{p}(t,\tau )\in \;B,\;G\;=\;g,\;S\;=\;s]\;\approx \;E[\hat{p}\;(t,\tau )\;|\;\hat{p}(t,\tau )\;\in B,\;G\;=\;g,\;S\;=\;s].$$

where B denotes a clinically meaningful risk interval. For longitudinal trajectories, the simulated distribution of future states should be compatible with empirical transition distributions observed in comparable patients:

$$\hat{F}(g,s,\tau ;\;Y)\;\approx \;F\_\mathrm{obs}(g,s,\tau ;\;Y).$$

In finite clinical datasets, population calibration should not be interpreted as requiring reliable empirical assessment in every possible subgroup intersection. Sparse subgroups, rare events, short follow-up, and irregular longitudinal measurements are practical limits to trajectory validation. Therefore, subgroup and trajectory-calibration analyses should be prespecified, restricted to clinically justified strata, reported with confidence or uncertainty intervals, and interpreted in relation to the effective sample size and number of observed events. When subgroup evidence is sparse, the appropriate conclusion may be that calibration is uncertain or not assessable, rather than that the twin is either valid or invalid. Partial-pooling, hierarchical modelling, shrinkage, and sensitivity analyses may help stabilize estimates, but they do not remove the need to report uncertainty transparently.

The framework therefore does not propose a universal statistical test for population calibration. Rather, it specifies a validity question: are the individualized outputs of the twin empirically credible for the population, subgroup, time horizon, and clinical decision for which they are being used? Operationally, population calibration should be assessed at prespecified landmark times, prediction horizons, and clinically relevant strata. For binary or time-to-event outcomes, this may involve horizon-specific calibration-in-the-large, calibration slopes, flexible calibration curves, observed-to-expected event ratios, and time-dependent Brier scores. For longitudinal or multistate trajectories, a practical diagnostic is to compare observed and simulated state occupancy, transition probabilities, or trajectory summaries within clinically meaningful strata, using uncertainty intervals and, where appropriate, distributional scores such as the continuous ranked probability score. The relevant diagnostic should be chosen according to the clinical claim made by the twin: absolute risk prediction, disease-state transition, treatment-response simulation, or care-pathway forecasting.

This definition has several implications. First, personalization does not exempt a model from population validation; rather, the credibility of an individualized forecast depends on whether similar individuals in similar settings experienced similar trajectories. Second, calibration is not limited to the baseline model-development dataset; it must be assessed temporally, geographically, and across clinically meaningful subgroups. Third, counterfactual outputs require an additional causal layer. If the twin estimates what would happen under alternative interventions, the estimand must be formulated in terms of potential outcomes, such as Yᵢ(ā), and evaluated using explicit assumptions about time zero, eligibility, treatment assignment, confounding, adherence, censoring, and competing events. In this sense, population calibration provides statistical credibility, whereas causal specification provides interpretability for simulated interventions. For time-to-event predictions in which competing events preclude the occurrence or observation of the outcome of interest, population calibration should be assessed on the clinically relevant risk scale, such as the cumulative incidence function, and the choice between cause-specific and subdistribution estimands should be aligned with the clinical claim (23).

Box 2 **Worked example: population-calibration audit of a published oncology digital twin.**

Chaudhuri et al. (38) developed a predictive digital twin framework for optimizing patient-specific radiotherapy regimens in high-grade glioma under uncertainty. The model combined a mechanistic tumour-growth and treatment-response model with population-informed parameter priors, Bayesian calibration using patient-specific magnetic resonance imaging data, and multi-objective risk-based optimization of radiotherapy schedules. In an in silico cohort of 100 patients, the personalized regimens were reported to increase median tumour time-to-progression by approximately 6 days for the same total radiation dose as standard-of-care radiotherapy, or to reduce the total radiation dose by 16.7% while maintaining a comparable level of tumour control. This makes the study a useful test case for the present framework because it moves beyond representation and prediction toward counterfactual and interventional claims.

Applying the claim-to-evidence framework shows that several credibility elements are present. The model is mechanistically specified; uncertainty is explicitly represented; population-level information is used to initialize prior distributions; patient-specific imaging data are used for Bayesian updating; and alternative treatment strategies are compared under uncertainty. However, the population-calibration evidence remains incomplete for clinical deployment. The reported outputs are not accompanied by external validation of predicted tumour trajectories against observed outcomes in an independent clinical population; there is no horizon-specific calibration curve comparing predicted and observed progression risk; subgroup calibration by clinically relevant factors such as age, sex, molecular subtype, tumour location, or treatment centre is not assessed; and the transportability of the model across imaging protocols, institutions, and treatment pathways is not demonstrated. Because the model recommends alternative radiotherapy regimens, its clinical claim is counterfactual or interventional rather than merely predictive, and would therefore require causal specification, prospective clinical evaluation, and post-deployment monitoring before being used to guide care.

**Table:** 

**Evidence domain**	**Evidence present in the published study**	**Evidence still needed for clinical deployment**
Clinical claim	Patient-specific radiotherapy optimization under uncertainty	Explicit intended-use statement: planning support, recommendation, or autonomous treatment optimization
Model type	Mechanistic tumour-growth and treatment-response model	Context-of-use-specific credibility assessment
Patient-specific updating	Bayesian calibration using patient-specific MRI data	Robustness across imaging schedules, segmentation methods, and scanner protocols
Population anchoring	Population-informed priors and in silico cohort reflecting reported glioma properties	External empirical calibration against observed trajectories in an independent clinical cohort
Uncertainty	Uncertainty-aware optimization and risk-based decision-making	Calibration of uncertainty intervals and coverage probabilities in external data
Trajectory calibration	Simulated tumour growth and treatment-response trajectories	Observed-vs.-predicted trajectory diagnostics at prespecified horizons
Subgroup calibration	Not demonstrated	Assessment by molecular subtype, age, sex, tumour location, treatment centre, and care pathway where sample size allows
Counterfactual validity	Alternative radiotherapy schedules simulated	Explicit causal estimand, assumptions about treatment assignment, adherence, toxicity, competing risks, and clinical decision rules
Clinical utility	In silico improvement in tumour-control/dose trade-off	Prospective clinical-impact study, safety evaluation, workflow assessment, and post-deployment monitoring

Population calibration has three functions. The first is statistical. Individual-level predictions should be calibrated against observed event rates in populations that resemble the intended-use setting. This includes calibration-in-the-large, calibration slope, flexible calibration curves, temporal validation, and subgroup calibration. The second function is clinical. A twin should represent not only biological variables, but also comorbidity, frailty, treatment access, adherence, health behaviors, and competing risks, all of which influence the real-world effect of a decision. The third function is ethical. If model performance differs across age, sex, socioeconomic status, ancestry, disability, or health-system context, the digital twin may reinforce inequity despite high aggregate accuracy (24). Fairness assessment should therefore be prespecified and metric-specific. Depending on the claim and deployment context, relevant diagnostics may include calibration within groups, error-rate balance, equality of opportunity, equalized odds, individual fairness, and assessment of proxy outcomes that may encode structural inequities. These criteria are not interchangeable and may be mutually incompatible when outcome prevalences or access to care differ across groups; therefore, the choice of fairness metric should be clinically and ethically justified rather than treated as a generic model-performance appendix (25–28).

This is particularly relevant for preventive medicine. Digital twins are often described as tools for proactive care, but prevention requires reliable absolute risk estimation over time, not only state reconstruction. A prevention-oriented twin must know whether a simulated trajectory is plausible for a patient embedded in a specific population, care pathway, and environment. Cohorts, registries, electronic health records, claims data, biobanks, and connected-device data should therefore be used not only as training sources, but also as external reference distributions for calibration, transportability assessment, and updating. In this sense, a clinically trustworthy twin is not a purely individualized artefact. It is an individual model disciplined by population evidence.

Population calibration should not be understood as a re-labelling of external validation, subgroup calibration, or transportability assessment. External validation evaluates model performance in data not used for model development; subgroup calibration evaluates agreement between predicted and observed outcomes within prespecified groups; and transportability assesses whether model performance is preserved across settings, time periods, devices, or populations (17, 29, 30). Population calibration adds a distinct claim-level constraint for digital twins: the patient-specific trajectories and scenario-based simulations generated by the twin should remain compatible with the empirical joint distribution of outcomes, transitions, treatment patterns, competing events, and care pathways in the intended-use population (31, 32) (Pash et al. is a preprint and was not peer reviewed). In this sense, population calibration is not a new estimator, but an evidentiary architecture that anchors individualized simulation in population-level empirical reality.

This distinction is important because a digital twin can satisfy conventional external validation for a prediction task while still producing implausible trajectories when used dynamically, longitudinally, or counterfactually. For example, a model may be well calibrated for 5-year cardiovascular risk in an external cohort, yet generate unrealistic simulated benefits if treatment adherence, competing mortality, frailty, or access to care are not aligned with the population in which the twin is deployed (29–31). Population calibration therefore shifts the focus from the average performance of an algorithm to the clinical credibility of the full distribution of simulated futures produced for a patient embedded in a specific population and care system (31–33).

A further distinction is needed between data-driven/statistical twins and mechanistic or biophysical twins. Population calibration is most directly applicable to digital twins that learn empirical relationships from observed clinical, biological, or health-system data and then estimate risks, trajectories, treatment responses, or care-pathway outcomes in an intended-use population. For these models, the credibility of an individualized forecast depends substantially on whether predicted trajectories are compatible with outcomes observed among comparable individuals in comparable settings. However, mechanistic and biophysical twins rest on a partly different validity argument. Their distinctive value may lie precisely in extrapolating to individuals, parameter regimes, or intervention scenarios for which no directly comparable population exists. In such cases, credibility should not be judged by population calibration alone, but by biophysical plausibility, parameter identifiability, sensitivity analysis, verification, validation against relevant experimental or clinical observations, uncertainty quantification, and an explicitly defined context of use (33–37).

### A validation framework for clinically trustworthy twins

**Figure 1:**
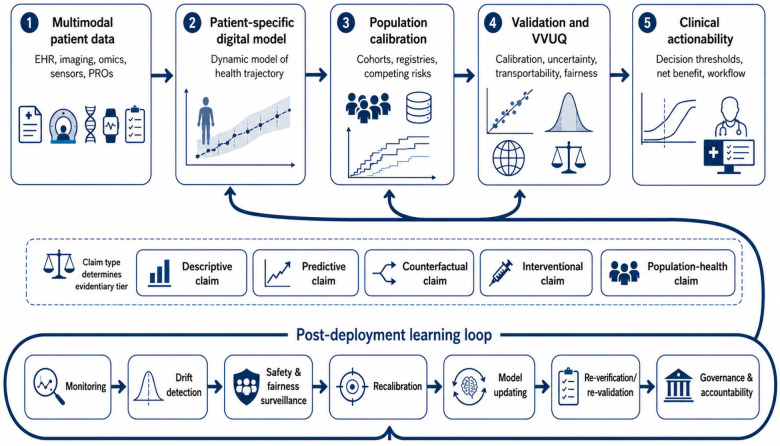
Validation and implementation pathway for clinically trustworthy digital twins. A clinically trustworthy digital twin integrates multimodal patient data into a patient-specific model, but its predictions and simulations should be constrained by population-level calibration. The framework distinguishes model construction from validation, causal interpretation, and implementation. Continuous post-deployment monitoring is required to detect data drift, calibration drift, subgroup performance degradation, safety concerns, and changes in clinical utility. EHR, electronic health record; PRO, patient-reported outcome; VVUQ, verification, validation, and uncertainty quantification.

The proposed framework is shown in Figure 1. It begins with multimodal patient data, including electronic health records, imaging, biomarkers, omics, sensors, patient-reported outcomes, and environmental exposures. These data inform a patient-specific digital twin that represents the current health state and plausible future trajectories. The distinctive feature of the framework is the population calibration layer, which anchors individual predictions in real-world incidence, transition probabilities, treatment patterns, competing risks, and subgroup heterogeneity. This layer is followed by validation, causal interpretation, and implementation, each of which corresponds to a distinct methodological question (Box 2).

Verification and validation should be treated as separate tasks. Verification asks whether the computational implementation correctly solves the mathematical, statistical, or mechanistic model. Validation asks whether the model is adequate for its intended clinical purpose. Recent work on verification, validation, and uncertainty quantification in digital twins for precision medicine has emphasized that credibility cannot be established without systematic assessment of model form, input uncertainty, parameter uncertainty, numerical error, and decision uncertainty (33). These principles should be brought into routine digital health evaluation.

For predictive twins, the minimal evidence should include transparent reporting, risk-of-bias assessment, internal validation, external validation, calibration, and clinical utility assessment. TRIPOD + AI and PROBAST + AI provide relevant methodological anchors for prediction models using regression or machine learning (15, 16). Their relevance to digital twins is not limited to models explicitly described as prediction algorithms. Any digital twin that forecasts risk or trajectory is, at least in part, a prediction model. It should therefore be reported and appraised as such.

For counterfactual twins, prediction validation is necessary but insufficient. Simulating alternative interventions requires causal structure. The target trial emulation framework is useful because it forces investigators to specify eligibility, assignment strategies, time zero, follow-up, outcomes, causal contrasts, and analysis plans before using observational data (18). A digital twin that claims to compare treatment strategies should make these assumptions explicit, describe how time-varying confounding was addressed, and quantify sensitivity to unmeasured confounding. Without this step, counterfactual outputs risk being interpreted as treatment effects when they are only associations.

For interventional twins, the validation pathway should extend beyond retrospective performance. Early live evaluation can follow DECIDE-AI, while randomized trials or pragmatic impact studies can be reported using CONSORT-AI and designed using SPIRIT-AI when the intervention includes an AI component (19–21). In addition, the FUTURE-AI consensus guideline provides a broader lifecycle view of trustworthy and deployable AI, including fairness, universality, traceability, usability, robustness, and explainability (22). These principles are directly applicable to digital twins because the twin is not only a model; it is a socio-technical system embedded in clinical decision-making.

### Implementation, monitoring and governance

A clinically trustworthy digital twin should be evaluated after deployment. Real clinical environments are dynamic. Patient populations change, coding practices evolve, devices are replaced, treatment protocols shift, and disease patterns are disrupted by external events such as epidemics. These changes may produce data drift, calibration drift, or fairness drift. Evidence from medical machine learning shows that data drift can degrade performance and that model monitoring cannot be reduced to periodic reporting of discrimination alone (39–42). Digital twins are especially vulnerable to drift because they rely on continuous or repeated data flows. A corrupted sensor stream, a change in laboratory assay, or a modified imaging protocol can alter the state of the twin without obvious failure at the user interface.

Post-deployment governance should therefore include algorithmovigilance, defined here as the continuous post-deployment surveillance of algorithmic systems to detect data drift, calibration drift, performance degradation, safety signals, inequitable subgroup performance, inappropriate use, and unintended downstream consequences. This involves monitoring input distributions, missingness patterns, calibration, uncertainty, subgroup performance, clinician interaction, alert burden, override behaviour, and downstream outcomes. Recalibration and updating should be pre-specified rather than improvised after performance decay. This lifecycle view is also increasingly aligned with regulatory requirements. In the European Union, AI-enabled systems used for clinical decision support, triage, prioritization, diagnosis, monitoring, or treatment recommendation may fall within the EU AI Act when they qualify as high-risk AI systems (43), particularly when the AI system is itself a product, or a safety component of a product, covered by Union harmonisation legislation and subject to third-party conformity assessment. The AI Act requires high-risk AI systems to be supported by risk management, data governance, technical documentation, record keeping, transparency, human oversight, accuracy, robustness, cybersecurity, conformity assessment, and post-market monitoring. In parallel, software that has a medical purpose may fall under the Medical Device Regulation or the *in vitro* Diagnostic Medical Device Regulation, with specific requirements for qualification, classification, clinical or performance evaluation, post-market surveillance, vigilance, and significant changes (44, 45). In the United States, the FDA framework for predetermined change control plans for AI-enabled device software functions formalizes the principle that planned modifications, the methodology for developing and validating those modifications, and their impact assessment should be specified prospectively (46). Therefore, the recommendation that recalibration, updating, and monitoring be pre-specified should be understood not only as a methodological safeguard but also as part of an emerging regulatory lifecycle for clinically deployed digital twins and AI-enabled medical software.

Governance should also define accountability. Clinicians need to know whether the twin is providing visualization, risk estimation, causal simulation, or treatment recommendation. Patients need to know how their data are used, whether predictions are uncertain, and whether outputs have been clinically validated for people like them.

Several examples illustrate why population calibration changes the evidentiary standard for digital twins. In cardiovascular prevention, a twin may integrate age, sex, blood pressure, lipids, diabetes, smoking, coronary calcium, electrocardiography, medication history, physical activity, sleep, and repeated biomarker measurements to forecast an individual's risk of myocardial infarction or heart failure. If used only to visualize longitudinal risk factors, the twin remains descriptive. If it estimates absolute 5- or 10-year cardiovascular risk, it becomes predictive and must be calibrated against observed event rates in comparable populations, including women, older adults, patients with multimorbidity, and patients treated in different health systems. If it simulates the effect of initiating statins, antihypertensive therapy, weight loss, or exercise, it becomes counterfactual and requires causal assumptions about treatment assignment, adherence, competing mortality, and time-varying confounding. If it recommends treatment intensification, it becomes interventional and requires prospective evidence of clinical utility, safety, and workflow acceptability (7, 15–18, 22, 33).

In oncology, a digital twin may combine tumour imaging, histology, molecular data, circulating biomarkers, treatment exposure, and longitudinal response measures to simulate tumour growth or treatment response. A mechanistic model that reconstructs tumour dynamics from repeated imaging may be scientifically valuable, but its clinical claim changes when it is used to compare radiotherapy schedules, treatment delay, immunotherapy combinations, or surveillance intervals. In that setting, uncertainty quantification is essential because imaging frequency, measurement error, segmentation uncertainty, model identifiability, and biological heterogeneity can all alter the simulated trajectory. Population calibration would require the patient-specific tumour trajectory to remain compatible with empirical distributions of progression, response, toxicity, and survival observed in comparable patients receiving comparable care. Otherwise, a visually persuasive tumour simulation could overstate the precision of individualized treatment planning (33, 34).

A third example concerns population-health and hospital-operation twins. A hospital twin may simulate emergency department crowding, bed demand, discharge capacity, staffing constraints, or downstream effects of screening programmes. Such a system may be useful for planning even if it does not directly recommend a treatment for a specific patient. However, if it is used to prioritize patients, allocate scarce resources, defer admissions, or trigger automated operational decisions, it becomes an interventional or population-health twin. Its evaluation should therefore include calibration of predicted demand, temporal validation across seasons and external shocks, fairness assessment across patient groups, and monitoring for unintended consequences such as delayed care, selection bias, or workload displacement. This example shows that the same population-calibrated logic applies beyond organ-level or patient-level twins: the unit of twinning may be a patient, a disease trajectory, a care pathway, or a health system, but the evidentiary standard must still match the decision being influenced.

Implementation should not be treated as the final stage after technical success. It should be integrated from the beginning. A twin that cannot be interpreted, trusted, or acted upon will not improve care even if it is statistically accurate. Conversely, a well-calibrated model may cause harm if it produces excessive alerts, shifts responsibility without governance, reinforces existing inequities, or encourages automation bias. Clinical usefulness should therefore be assessed through decision-curve analysis and net benefit, change in management, patient-relevant outcomes, safety signals, cost-effectiveness, and acceptability (47). In digital health, a model is not clinically successful because it is deployed; it is successful when deployment improves decisions without producing disproportionate harms.

### Perspective: toward epidemiologically grounded digital health

The field of medical digital twins is now mature enough to move beyond conceptual enthusiasm. The next scientific step is not simply to build larger models, integrate more modalities, or increase computational fidelity. It is to define the evidentiary standards that connect a model to a clinical claim. Population calibration provides one route toward this goal because it connects individual simulation to the empirical constraints of real patients, real trajectories, and real care systems.

This perspective also reframes the relationship between personalized medicine and public health. Personalized digital health cannot be credible if it ignores the population distributions from which individuals arise. Similarly, public health cannot become more precise if it does not incorporate longitudinal, patient-level heterogeneity. Population-calibrated digital twins may therefore provide a bridge between individualized prevention and learning health systems. They could support early risk stratification, adaptive follow-up, treatment optimization, and resource planning, provided that their validation matches their clinical ambition.

Several research priorities follow. Digital twin studies should pre-specify the clinical claim made by the model. They should distinguish representation, prediction, counterfactual simulation, and intervention. They should report calibration and uncertainty as primary evidence rather than technical appendices. They should test transportability across sites, time periods, devices, and subgroups. They should link counterfactual claims to explicit causal designs. Finally, they should treat implementation, monitoring, and updating as part of the model lifecycle, not as optional *post hoc* activities. These priorities are demanding, but they are proportional to the risks created when simulations enter clinical decision-making.

## Conclusion

Medical digital twins will not become clinically trustworthy because they are more complex. They will become trustworthy if their clinical claims are matched by appropriate evidence. A clinically trustworthy digital twin should be empirically anchored in the intended-use population, statistically calibrated, uncertainty-aware, causally explicit when it simulates interventions, transportable across settings, equitable across populations, and embedded in a monitored clinical workflow. Without this methodological foundation, digital twins risk remaining impressive but fragile simulations. With it, they could become a robust infrastructure for personalized prevention, adaptive treatment, and digitally enabled learning health systems. The label “digital twin” should neither dilute established standards for prediction, simulation, causal inference, or clinical decision support, nor be used to imply clinical trustworthiness before the relevant evidence has been produced.

## Data Availability

The original contributions presented in the study are included in the article/Supplementary Material, further inquiries can be directed to the corresponding author/s.
